# Waitlist management in a pediatric weight management clinic: implementing an orientation session

**DOI:** 10.1186/s12887-021-02868-w

**Published:** 2021-09-22

**Authors:** Webb A. Smith, Emily Gray, Tamekia L. Jones, Joan C. Han, E. Thomaseo Burton

**Affiliations:** 1 Department of Pediatrics, College of Medicine, University of Tennessee Health Science Center, 50 North Dunlap Street, Room 453R, TN 38103 Memphis, USA; 2 Children’s Foundation Research Institute, Le Bonheur Children’s Hospital, 50 North Dunlap Street, Room 453R, TN 38103 Memphis, USA; 3 Department of Health Promotion and Disease Prevention, College of Nursing, University of Tennessee Health Science Center, TN Memphis, USA; 4 Department of Preventive Medicine, College of Medicine, University of Tennessee Health Science Center, TN Memphis, USA; 5 Department of Physiology, College of Medicine, University of Tennessee Health Science Center, TN Memphis, USA

**Keywords:** Pediatric obesity, Treatment initiation, Engagement, Motivation, Attrition, Referral

## Abstract

**Background:**

This study evaluates implementation of an orientation session to address a waitlist of more than 2000 referrals to a pediatric weight management clinic in the Mid-South United States.

**Methods:**

An hour-long group-based orientation to the pediatric weight management clinic was implemented to provide information about the structure and expectations of the clinic as well as education on healthy lifestyle recommendations. Families were contacted from the waitlist by telephone and invited to attend an orientation session prior to scheduling a clinic appointment.

**Results:**

Of 2251 patients contacted from the waitlist, 768 scheduled an orientation session, of which 264 (34 %) attended. Of the 264 orientation participants, 246 (93 %) scheduled a clinic appointment. Of those, 193 (79 %) completed a clinic visit. Waitlist times decreased from 297.8 ± 219.4 days prior to implementation of orientation sessions to 104.1 ± 219.4 days after.

**Conclusions:**

Orientation has been an effective and efficient way to triage patient referrals while maximizing attendance in limited clinic slots for patients and families demonstrating interest and motivation. Elements of this approach are likely generalizable to other pediatric clinical settings that must strategically manage a large volume of patient referrals.

## Background

The prevalence and severity of pediatric obesity have risen dramatically over the past several years, particularly among racial/ethnic minority youth [1, 2]. Children and adolescents with obesity are at increased risk for physical (e.g., hypertension, type 2 diabetes) and socioemotional (e.g., depression, anxiety, stigmatization) comorbidities as well as premature mortality, all of which are magnified as severity of obesity increases [3, 4]. The high rates of prevalence coupled with the high burden of disease in both financial costs and impact on quality of life make management of pediatric obesity and related health conditions earlier in their trajectory a key public health priority.

Comprehensive management of pediatric obesity may be outside the scope of general practitioners, who are therefore encouraged to refer severe and refractory cases for tertiary interdisciplinary care [5, 6]. However, demand for these comprehensive programs tends to outpace the clinical capacity for treatment [7, 8]. In fact, a survey of 24 hospital-based pediatric weight management clinics revealed an average wait time of two months for an initial appointment, with an upper range of 10–12 months [9]. Such supply and demand mismatches are quite problematic as long waits for treatment have been linked to decreased engagement and adherence and increased nonattendance and attrition [10, 11].

Despite prevailing medical concern, not all patients and families are appropriate referrals for comprehensive pediatric obesity care. Interdisciplinary clinics require significant investments of time, money, and personnel [6], and efforts to maximize these investments are of paramount importance [12]. Hindrances to patient participation include logistical challenges, ambivalence around making lifestyle (i.e., dietary, exercise) changes, and lack of motivation to attend frequent appointments [9, 11–13]. Additionally, some patients and families may wish to implement healthy lifestyle changes on their own while others do not consider obesity to be a problem [14, 15]. Finally, common predictors of non-initiation with pediatric weight management include public insurance, race/ethnicity, and low-income status [15, 16], which may actually point to competing priorities wherein families make healthcare decisions in a context of limited time and resources [17].

The unmet supply of comprehensive pediatric obesity programming is exacerbated by disinterest and amotivation in a subset of patients as well as waning motivation among patients and families who may have been ready to make changes at the time of initial referral [15, 18]. As such, it is important to direct existing resources to those most ready to take advantage of pediatric weight management [19]. The purpose of this project was to evaluate the implementation of an orientation session within a pediatric weight management clinic. The threefold aims of the orientation were to:

1. Actively manage a waitlist of newly referred patients;
2. Triage newly referred patients for more immediate treatment based on interest and readiness to engage in lifestyle changes;
3. More immediately introduce healthy lifestyle principles to newly referred patients.

## Methods

The present study is an examination of aggregate-level data and does not involve individually identifiable or protected health information. The study was reviewed and deemed exempt by the University of Tennessee Health Science Center Institutional Review Board.

The Healthy Lifestyle Clinic (HLC) was established to manage pediatric obesity and comorbid conditions in the Mid-South, a traditionally underserved region of the United States [20]. The HLC began accepting patient referrals in the summer of 2014 based on standardized criteria: Body Mass Index (BMI) ≥ 95th percentile for age and sex or ≥ 85th percentile with at least one comorbidity (e.g., obstructive sleep apnea, hypertension, type 2 diabetes). Referrals were also required to include documentation of physical evaluation by a primary care or specialty provider.

Upon receipt of referrals, patients were scheduled into intake appointment blocks that entailed administration of surveys on health behaviors and medical history, anthropometric and laboratory measurements, and individual evaluations with members of the interdisciplinary team (i.e., medicine, nutrition, behavioral health, exercise). Notably, due to the nature of the referral process, it could not be assumed that patients and families were informed about the reason for their referral. As such, it became critical to provide referred patients a baseline level of information about the HLC in order to facilitate their decision about participating in the clinic.

New patients were scheduled concurrently, with capacity to see 3–5 patients in a 5-hour clinic. As referrals mounted, a waitlist for HLC appointments developed. In fact, within two years, there were more than 1700 patients waiting for an initial appointment. However, the actual number of patients seen was only moderate due to last-minute cancellations and no shows, which led to frustration by HLC providers, referring physicians, and potential patients.

### Addressing the Waitlist

Resolving the referral backlog required a strategic contact plan to ensure that each potential patient had ample opportunity to participate in the HLC. Since some of the referred patients had spent a year or more waiting for an intake appointment, due diligence was necessary to make certain that adequate contact was attempted before determining disinterest and removing them from the waitlist. In the fall of 2016, HLC staff began the process of contacting waitlisted patients.

Patients on the waitlist were contacted through telephone calls made by clinic administrative staff. A database was created using research electronic data capture (REDCap; [21]) to log dates, status, and disposition of individual patient referrals as well as document contact attempts. Specifically, the database was used to generate up-to-date call lists to reflect the latest attempted contact of waitlisted patients.

Each referred patient on the waitlist could receive up to three phone calls. For each call, date, time, and disposition (i.e., interested in HLC, not interested in HLC, bad contact, no answer) were recorded in the REDCap database to track patients from referral to resolution. The first phone call occurred during standard business hours while subsequent calls were made during evening hours. Calls that resulted in no answer received a generic voice mail stating, “This is the Healthy Lifestyle Clinic. Please give us a call at (xxx) xxx-xxxx when possible.” These patients remained on an active call list and received additional calls within a few days. After the third phone call, the referring physician was sent a letter informing them of the multiple failed attempts at contact. For those referrals with incorrect or out-of-service phone numbers, a letter was generated to the referring physician requesting updated contact information. Patients expressing disinterest in the clinic were removed from the waitlist and their referring physician informed by letter that the patient declined to participate in the HLC. It is important to note that patients declining to participate were offered the option to be contacted at a later date and assured that they were welcome to initiate contact at any time should their interest in seeking care at the HLC change. Finally, patients expressing interest in the HLC were scheduled to attend an orientation session.

### The Orientation Session

In November 2016, the HLC orientation was implemented as a first point of contact for waitlisted and subsequent newly referred patients. Each hour-long group-based information session accommodates up to 50 potential patients and their families and provides a detailed overview of the clinic (see Table 1). Orientation sessions are scheduled on weekday evenings (5:00pm) and Saturday mornings (11:00am) and the format entails a PowerPoint presentation led by an HLC provider. The intent of each session is to facilitate patients and their families in making an informed decision about whether to participate in the HLC. Specifically, the presentation provides a rationale for addressing obesity early in its trajectory, addresses the focus of the clinic on overall health (as opposed to a sole focus on weight), and introduces the interdisciplinary team that works together to support patients and their families. Throughout the presentation, families are positioned as integral components of the care team.

**Table 1:** Outline of Orientation Session Topics

I. Why are We Concerned about Childhood Obesity?
a. Medical Comorbidities
b. Psychological Health
c. Social Functioning
II. Definition of Key Terms
a. Overweight
b. Obesity
c. Body Mass Index (BMI)
III. Measuring Success in the Healthy Lifestyle Clinic
a. Reductions in Weight / Body Fat
b. Management of Medical Comorbidities
c. Setting and Working Towards Individual and Family Goals
d. Engagement in Activities of Daily Living
IV. A Team-Based Approach: Interdisciplinary Components
a. Medicine
b. Behavioral Medicine
c. Nutrition
d. Exercise
V. Clinic Expectations of Patients and Families

Inculcation of healthy lifestyle principles begins in the orientation session by way of an interactive quiz that is interspersed throughout the presentation. Quiz questions are patterned after the 5-2-1-0 Let’s Go! public health campaign [22] which encourages ≥ 5 servings of fruits and vegetables per day, ≤ 2 h of non-school related screen time per day (updated to ≤ 1 h per 2016 American Academy of Pediatrics recommendations [23]), ≥ 1 h of physical activity daily, and avoidance of sugary drinks (see Table 2). An additional question addresses age-based sleep requirements [24]. Using a remote response system (TurningPoint, Youngstown, OH), patients enter their answer to each question and receive real-time feedback. Each question is followed by a brief discussion on rationale for the guidelines and practical ways to implement the guidelines into daily life.

**Table. 2:** Orientation Questions

Topic	Question	Educational Highlights
Sleep	a) True or False: 8 h is the perfect amount of sleep for every child.	• Sleep hygiene • Sleep needs vary by age
Fruit and Vegetable Intake	b) It’s recommended that we eat 5 servings of fruits and vegetables every _____.	• Don’t be fooled by packaging • Fruits and vegetables come in many forms, including frozen (costs, convenience)
Electronic Screen Time	c) It is recommended that children have no more than _____ hour(s) of non-school related screen time each day.	• Sedentary time hinders physical activity • Exposure to advertisements can be problematic
Exercise	d) Josefina and Hector are twins. Josefina likes to go for an hour-long jog everyday with her mom. Hector has gym class every morning for 30 min and he likes to ride bikes with his friends after school for about 30 min. Which twin is meeting the daily exercise recommendation?	• Exercise is incremental • Vigorous PA is characterized by increased heart rate, breath rate, and sweating
Sugary Drink Intake	e) How many sugary drinks should we have each day?	• Many beverages contain sugar, even juices • Increase intake of water

Answers: (a) false; (b) day; (c) one; (d) both; (e) zero

The final component of the orientation session is a detailed review of what to expect at the first clinic visit (e.g., paperwork, bloodwork, visits with interdisciplinary providers) as well as reminders of how to prepare for the appointment (e.g., plan to be on time, bring medications and prescriptions for reconciliation, wear comfortable clothes and appropriate shoes for exercise). This is followed by discussion of interest in scheduling an intake appointment in HLC. Each potential patient returns a card with updated contact information and indication of interest in the clinic. Those interested are contacted by clinic staff to review insurance and appointment options within 1–2 business days. Those who are not interested are provided information regarding other community-based resources for management of pediatric obesity, and their referring physician informed by letter that the patient declined to participate in the HLC.

## Results

Between July 2014 and November 2016, the HLC received 3067 new patient referrals from primary care providers or other pediatric subspecialists; referrals continued to accrue at a rate 36.3 ± 8.5 new patients per month. As of November 2016, when the orientation program was instated, the HLC waitlist included 1749 potential patients. By November 2017, only 66 patients, all of whom were referred within the previous 45 days, were awaiting first contact. Currently, the HLC receives between 40 and 50 new patient referrals per month with most referrals being contacted within 30 days. The clinic has no waitlist; the majority of patients are scheduled into an orientation session within 2 weeks and an HLC intake appointment within 30 days of referral.

### Managing the Waitlist

**Fig. 1:**
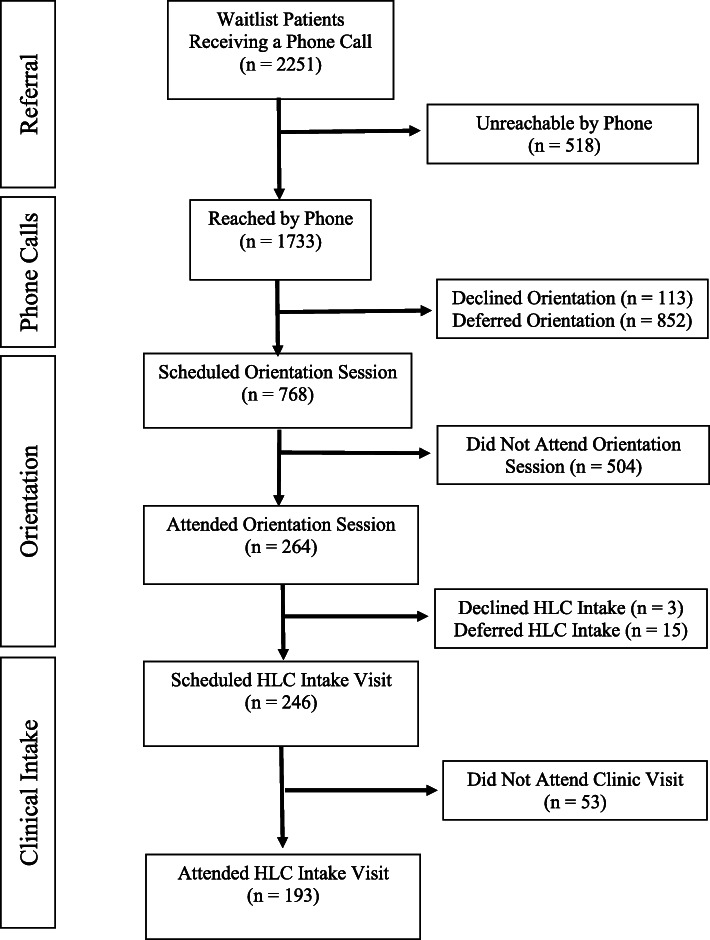
Flow Diagram of Healthy Lifestyle Clinic Referrals to Intake Visit

To achieve this abovementioned improvement between November 2016 and November 2017, 2251 patients from the actively accruing waitlist were called and invited to attend the HLC orientation. As shown in Fig. 1, these attempts resulted in contact of 34.4 % (n = 776) of families upon the first phone call during normal business hours and an additional 37.5 % (n = 844) were contacted on a subsequent phone call outside of normal business hours. A small number (113; 5.1 %) of referrals, when reached by phone, expressed disinterest in the HLC. Ultimately, more than a fifth of referred patients (n = 518, 23.0 %) were unreachable by phone, which prompted a letter to the referring provider communicating the HLC’s inability to contact the family.

Of the families reached by phone calls, 768 (44.3 %) were scheduled for an orientation session during that call. Those who did not schedule immediately were instructed to call back when they were ready to make an appointment. Of those who were scheduled for an orientation, 264 (34.4 %) actually attended the session. Of those 264 orientation participants, 246 (93.2 %) expressed interest in scheduling an HLC appointment. Three families were not interested after attending orientation and 15 families requested to delay setting an HLC intake appointment in favor of a more convenient time (e.g., school vacation). For those who agreed to schedule an HLC intake appointment session, 193 (78.5 %) completed an intake visit. While only 8.6 % of phone calls were converted to clinic intake appointment, this represents a 35 % increased attendance rate from the same period of time before beginning the orientation sessions. In addition to the improved clinic attendance rate, the mean (± standard deviation) number of days on the waitlist decreased from 297.8 ± 219.4 days prior to implementation of orientation sessions to 104.1 ± 219.4 days after.

HLC provider feedback emphasized that patients who attended an orientation session tended to express a greater awareness of the purpose of the clinic. Additionally, patients and their families seemed to be more aware of what to expect during their first visit, particularly the length of the appointment. Finally, discussions with many families indicated that they had implemented healthy behaviors in the interim period between orientation and their first appointment.

## Discussion

Considering the increasing prevalence and severity as well as the short- and long-term sequelae associated with pediatric obesity, timely intervention is essential. Unfortunately, many tertiary care clinics are unable to meet the overwhelming demand due to limited capacity and difficulty engaging patients and their families in treatment [25]. While a number of pediatric weight management clinics have attempted to address logistical concerns and demographic characteristics that predict engagement and attendance patterns [26–28], the present study examined a clinic initiative to triage patient referrals while prioritizing limited clinic slots for patients and families demonstrating interest and readiness to engage in lifestyle changes to address excess weight. Results indicate that efforts to address a backlog of patient referrals were efficacious and efficient. In a year’s time, more than 2000 individual patient referrals were contacted and dispositioned, which included investigating outdated contact information. Rather than allowing patients to linger on a waitlist until clinic slots became available, an intermediary orientation program was introduced.

Orientation served as an opportunity to contact referrals quickly, thereby capitalizing on the recency of their motivation to engage in health behavior change. In line with qualitative research findings on caregivers’ recommendations to enhance enrollment in pediatric weight management [15, 29], patients and families were provided information about the structure and expectations of the HLC, thus allowing them to make an informed decision about seeking care. Orientation sessions, which have been shown to identify patients and families who are ready to engage in weight-related behavior change, have been implemented to address attrition from pediatric weight management programs [30]. However, potential patients and families must first engage with weight management before outcomes such as adherence and attrition can be assessed [25]. As such, the present study focused on conversion of referrals to enrolled patients.

Immediately following implementation of the orientation sessions, show rates for the HLC improved notably and the amount of time referred patients spent on the waitlist reduced drastically. In fact, the average length of wait was almost three times shorter during the year following implementation of orientation. Research on attendance patterns after pediatric subspecialty referrals has shown that decreasing the wait from referral to initial appointment is associated with improved visit attendance [31]. Relatedly, pressure on the front desk staff to repeatedly describe why patients were referred, length of appointments, and how the clinic works was allayed. These outcomes represent notable improvements over traditional referral handling and scheduling where patients were given the next available appointment without first screening for interest [19, 30].

Referral rates and the number of potential patients exceeded and continue to exceed the number of available HLC appointment slots. Consistent with previous research [29, 32], it was noted that during the course of contacting potential patients, many referred patients were either not interested in behavior and lifestyle intervention or were unaware of the reason for their referral. Implementation of the orientation sessions served as a triage system by which interested and motivated patients were able to schedule and attend clinic appointments while those who were not ready to engage were able to actively or passively communicate their interest in pediatric weight management.

In addition to waitlist management, the orientation session was an opportunity to provide initial education regarding standard health behavior changes associated with improved obesity-related outcomes. During the session, several questions are presented with the intention of generating discussion about health and wellness among family members and HLC providers. While these questions are not a formal evaluation of health knowledge, they have consistently stimulated conversation about sleep habits, fruit and vegetable intake, physical activity levels, sugary drink consumption, and electronic screen time. These questions have proven to be a valuable opportunity to engage families and introduce health and wellness counseling and are a key chance to set the stage for our family-based approach and the type of information that will be discussed during clinic visits. Furthermore, HLC providers have commented that some patients and their families have been able to implement these guidelines independently, thereby taking steps towards a healthier lifestyle before formally engaging with the HLC. Formal examination of pre-treatment implementation of lifestyle changes will be an important area of future research.

The results of this study should be considered in the context of the present limitations. Referred patients were contacted by phone to schedule appointments and discuss offerings of the HLC. This could have led to inadvertent exclusion of a subset of patients, particularly those without reliable phone service, a well-documented concern due to transiency of low-income populations [33–35]. Secondly, fewer than 10 % of phone calls to patients on the waitlist were converted to clinic intake appointments. It is important to consider this finding in light of the variable time patients spent on the waitlist; some patients had been referred more than a year prior to receiving a phone call while others were contacted within days of referral. It is also important to acknowledge that once the backlog was addressed, referrals were processed much more efficiently. Nevertheless, this finding underscores the importance of appropriate referrals.

Drawing on tenets of motivational interviewing, Ball and colleagues [19] created the *Readiness and Motivation Interview for Families* to aid healthcare providers in better understanding family-level motivational factors (e.g., perceived importance of lifestyle changes, perceived ability to implement lifestyle changes) that are so important to engagement and participation in treatment. Use of such measures may facilitate referral of patients and their families who are ready and willing to initiate and participate in pediatric weight management. Patients who are not ready to engage may benefit from less intensive support in the primary care setting or within their communities. In the HLC, implementation of high capacity orientation sessions allowed for engagement with patients and families across a wide range of motivations while ultimately mitigating loss of more limited clinical resources.

## Conclusions

While implementing an orientation session has been effective at triaging limited clinical resources for those demonstrating interest and motivation to take advantage, it continues to be important to try and reach patients and families with limited motivation to engage in pediatric weight management. Management of pediatric obesity requires commitment from all parties involved, including patients, family members, and providers. Further research is needed to acknowledge and examine social determinants that may hinder engagement, participation, and uptake of pediatric weight management resources. In particular, it will be important to assess utility of virtual platforms for engaging patients and families in pediatric weight management.

Experiences in the HLC highlight the overwhelming need for pediatric obesity treatment as well as the challenges of clinical obesity treatment, namely poor attendance and mixed motivation to make lifestyle changes. Results of this study provide insight into the importance of actively managing a waitlist of referred patients and managing the overwhelming numbers that accrued to efficiently utilize the limited clinical resources available; elements of this approach are likely generalizable to other pediatric clinical settings that must strategically manage a large volume of patient referrals.

## Data Availability

The datasets used and/or analyzed during the current study are available from the corresponding author on reasonable request.

## Abbreviations

U.S.: United States
HLC: Healthy Lifestyle Clinic
BMI: Body Mass Index
